# Sex differences in body fluid composition in humans with obstructive sleep apnea before and after CPAP therapy

**DOI:** 10.14814/phy2.15677

**Published:** 2023-04-20

**Authors:** David D. M. Nicholl, Patrick J. Hanly, Jennifer M. MacRae, Ann A. Zalucky, George B. Handley, Darlene Y. Sola, Sofia B. Ahmed

**Affiliations:** ^1^ Division of Nephrology, Department of Medicine, Royal Inland Hospital University of British Columbia Kamloops British Columbia Canada; ^2^ Division of Respirology, Department of Medicine, Cumming School of Medicine University of Calgary Calgary Alberta Canada; ^3^ Sleep Centre, Foothills Medical Centre Calgary Alberta Canada; ^4^ Hotchkiss Brain Institute, University of Calgary Calgary Alberta Canada; ^5^ Division of Nephrology, Department of Medicine, Cumming School of Medicine University of Calgary Calgary Alberta Canada; ^6^ Libin Cardiovascular Institute, University of Calgary Calgary Alberta Canada; ^7^ Alberta Kidney Disease Network Calgary Alberta Canada; ^8^ Department of Critical Care University of Calgary Calgary Alberta Canada; ^9^ Healthy Heart Sleep Company Calgary Alberta Canada

**Keywords:** bioimpedance, body composition, continuous positive airway pressure, obstructive sleep apnea, sex differences

## Abstract

Obstructive sleep apnea (OSA) is common in heart and kidney disease, both conditions prone to fluid retention. Nocturnal rostral fluid shift contributes to the pathogenesis of OSA in men more than women, suggesting a potential role for sex differences in body fluid composition in the pathogenesis of OSA, with men having a predisposition to more severe OSA due to an underlying volume expanded state. Continuous positive airway pressure (CPAP) increases intraluminal pressure in the upper airway and mitigates the rostral fluid shift; this, in turn, may prevent fluid redistribution from other parts of the body to the upper airway. We sought to determine the impact of CPAP on sex differences in body fluid composition. Twenty‐nine (10 women, 19 men) incident, sodium replete, otherwise healthy participants who were referred with symptomatic OSA (oxygen desaturation index >15/h) were studied pre‐ and post‐CPAP (>4 h/night × 4 weeks) using bioimpedance analysis. Bioimpedance parameters including fat‐free mass (FFM, %body mass), total body water (TBW, %FFM), extracellular and intracellular water (ECW and ICW, %TBW), and phase angle (°) were measured and evaluated for sex differences before and after CPAP. Pre‐CPAP, despite TBW being similar between sexes (74.6 ± 0.4 vs. 74.3 ± 0.2%FFM, *p* = 0.14; all values women vs. men), ECW (49.7 ± 0.7 vs. 44.0 ± 0.9%TBW, *p* < 0.001) was increased, while ICW (49.7 ± 0.5 vs. 55.8 ± 0.9%TBW, *p* < 0.001) and phase angle (6.7 ± 0.3 vs. 8.0 ± 0.3°, *p* = 0.005) were reduced in women compared to men. There were no sex differences in response to CPAP (∆TBW –1.0 ± 0.8 vs. 0.7 ± 0.7%FFM, *p* = 0.14; ∆ECW –0.1 ± 0.8 vs. −0.3 ± 1.0%TBW, *p* = 0.3; ∆ICW 0.7 ± 0.4 vs. 0.5 ± 1.0%TBW, *p* = 0.2; ∆Phase Angle 0.2 ± 0.3 vs. 0.0 ± 0.1°, *p* = 0.7). Women with OSA had baseline parameters favoring volume expansion (increased ECW, reduced phase angle) compared to men. Changes in body fluid composition parameters in response to CPAP did not differ by sex.

## Abstract 2

We utilized bio‐electrical impedance analysis to assess sex differences in body composition in 10 women and 19 men with obstructive sleep apnea (OSA) before and after treatment with continuous positive airway pressure (CPAP) therapy. We observed that despite similar total body water (% fat‐free mass), women with OSA had increased extracellular water and reduced intracellular water compared to men, suggestive of a volume expanded state, though the response of these body composition parameters to 1 month of CPAP therapy did not differ by sex. Our findings support that there are underlying sex differences in body composition in OSA, though contrary to our expectations, women with OSA in our study had body composition parameters which favored volume expansion compared to men. Importantly, the impact of CPAP therapy on body composition did not differ by sex.

## 1 INTRODUCTION

Obstructive sleep apnea (OSA) is common in congestive heart failure (CHF) (Javaheri et al., 1995) and chronic kidney disease (CKD) (Hanly & Pierratos, 2001; Nicholl et al., 2012; Wadhwa & Mendelson, 1992), both conditions prone to aberrant body composition and fluid retention. OSA is also associated with increased cardiorenal morbidity and mortality (Ahmed et al., 2011; Campos‐Rodriguez et al., 2012; Marti et al., 2002). Fluid retention and the nocturnal rostral fluid shift, whereby fluid from the lower extremities moves to the neck, contribute to the pathogenesis of OSA by promoting upper pharyngeal narrowing and worsening OSA severity (Elias et al., 2012; Gavrilovic et al., 2016; Kasai et al., 2012; Redolfi et al., 2009; Silva et al., 2017; Su et al., 2009). Continuous positive airway pressure (CPAP) therapy is an effective treatment for OSA (Epstein et al., 2009), which increases intraluminal pressure in the upper airway and mitigating the physiologic effects of the nocturnal rostral fluid shift (Silva et al., 2017), while promoting increases in lean body mass (Iftikhar et al., 2015; Münzer et al., 2010; Shechter et al., 2019).

Sex differences in the clinical presentation and pathogenesis of OSA are well established (O'Connor et al., 2000; Quintana‐Gallego et al., 2004; Valipour et al., 2007; Young et al., 1996). OSA disproportionately affects men (Lin et al., 2008; Quintana‐Gallego et al., 2004; Young et al., 1993), though women are frequently misdiagnosed or underdiagnosed due to differing OSA symptomatology compared to men, resulting in delayed initiation of treatment (Valipour et al., 2007; Young et al., 1996). Men with OSA require higher positive airway pressure for treatment of their OSA when compared to women with similar disease severity (Jayaraman et al., 2011), though CPAP promotes an increase in lean body mass and weight gain in both sexes (Drager et al., 2015; Münzer et al., 2010). However, differences in nocturnal fluid accumulation of the necks of women suggest a minor role of rostral fluid shift in the pathogenesis of OSA in women (Elias et al., 2012; Kasai et al., 2012), and therefore it is possible that CPAP therapy may impact body fluid composition by sex differently. Given recent endeavors to phenotype OSA and target treatment according to the predominant physiologic abnormality, a more comprehensive understanding of sex differences in body fluid composition in OSA and the response to CPAP therapy is needed.

Bio‐electrical impedance analysis (BIA) is a reliable, non‐invasive technique to measure body fluid and fat composition, based on the principle that electric current flows at different rates through the body depending upon its composition (Kyle et al., 2004a, 2004b). This allows for measurement of fat‐free mass (FFM) and total body water (TBW), along with its distribution into intracellular (ICW) and extracellular water (ECW) (Kyle et al., 2004b). As part of a study evaluating kidney function in humans with OSA (Nicholl et al., 2014), we conducted exploratory sex‐stratified analyses to evaluate whether the impact of CPAP therapy on body fluid composition differs between women and men with OSA. The rationale for our study can be summarized as follows. Nocturnal rostral fluid shift contributes more to the pathogenesis of OSA in men compared to women (Elias et al., 2012; Kasai et al., 2012). CPAP therapy increases intraluminal pressure in the upper airway which prevents fluid accumulation in the surrounding tissues in the neck (Epstein et al., 2009; Silva et al., 2017), which may prevent fluid redistribution from other parts of the body to the upper airway in men more than women. We hypothesized that (1) as men have a predisposition to more severe OSA, we expected that men would have increased ECW compared to women, indicative of a volume expanded state; and (2) as men are prone to more severe OSA from volume expansion, particularly through the nocturnal rostral fluid shift, we expected men to derive greater benefit from CPAP therapy with a reduction in ECW compared to women.

## 2 METHODS

### 2.1 Study participants

Women and men, age 18–70, with moderate to severe OSA and significant nocturnal hypoxemia (NH; defined later), were eligible to participate in the study. Participants were recruited from community patients referred for suspected OSA to the Foothills Medical Centre Sleep Centre and respiratory homecare companies (Healthy Heart Sleep Company, Dream Sleep Respiratory Services Ltd, and RANA Respiratory Care Company) in Calgary, AB, Canada, between 2011 and 2014, as part of a study evaluating kidney function in humans with OSA (Nicholl et al., 2014, 2020). All participants underwent a medical history, physical examination including anthropometric measurements, and laboratory screening. Exclusion criteria were based on the design of the original study (Nicholl et al., 2014). Briefly, exclusion criteria were cardiovascular, cerebrovascular, and kidney disease, uncontrolled hypertension (blood pressure [BP] >140/90 despite maximal use of 3 or more antihypertensive agents of different classes), diabetes mellitus, pulmonary disease, current treatment for OSA, current smoking, pregnancy, use of non‐steroidal anti‐inflammatory medications, or exogenous sex hormones.

The study protocol was approved by the Conjoint Health Research Ethics Board at the University of Calgary. Written informed consent was obtained from all study participants in accordance with the Declaration of Helsinki.

### 2.2 OSA assessment

Participants performed an unattended, home sleep apnea test (HSAT; Remmers Sleep Recorder [RSR] Model 4.2, Saga Tech Electronic) (Vazquez et al., 2000). The monitor consists of an oximeter to record oxyhemoglobin saturation (SaO_2_), a pressure transducer to record nasal airflow, a microphone to record snoring, and a body position sensor. The oximeter provides the data for an automated scoring algorithm, which calculates the oxygen desaturation index (ODI) based on the number of episodes of oxyhemoglobin desaturation ≥4%/h of monitoring. Nocturnal oxygen saturation was sampled at 1 Hz. The RSR has been validated by comparison to attended polysomnography (Vazquez et al., 2000).

OSA was defined as an ODI ≥15/h as this reflects moderate–severe sleep apnea which is likely to be clinically significant (Vazquez et al., 2000). The RSR has a sensitivity of 98% and specificity of 88% for an ODI ≥15/h (Vazquez et al., 2000). Portable monitoring was performed following current guidelines and recommendations (Collop et al., 2007). The data were reviewed by a sleep medicine physician (PJH) who confirmed that the estimated ODI was accurate and diagnostic of OSA. NH was defined as SaO_2_ <90% for ≥12% of the duration of nocturnal monitoring (Nieto et al., 2000). As Calgary is situated at an altitude of 1045 m a.s.l., we would expect SaO_2_ to be ~1.5% lower compared to sea level (Crapo et al., 1999).

### 2.3 Study protocol

Participants were instructed to consume >200 mmol/day of sodium for 3 days before each study day to ensure they were sodium replete. Participants were studied in the supine position in a temperature‐controlled, quiet room in the morning after an 8 h fast. All participants provided a second morning spot urine for determination of urinary sodium to confirm that participants were sodium replete (Kawasaki et al., 1993). All pre‐menopausal female participants were studied 14 days after the first day of the last menstrual period, determined by counting days and measuring 17β‐estradiol levels (Chidambaram et al., 2002). Menopause was defined as secondary amenorrhea ≥12 months. Participants on medications which alter kidney function and volume homeostasis were switched to the calcium‐channel blocker amlodipine to achieve adequate BP control 2 weeks prior to each study day (Seifarth et al., 2002). Amlodipine was taken daily each morning including the morning of the assessment. A standard BP cuff (Dinamap; Critikon) was placed on the right arm and the mean of two readings taken by the same registered nurse (DYS) were recorded. Mean arterial pressure was calculated as 1/3 systolic BP (SBP) + 2/3 diastolic (DBP). Laboratory measurements were taken as part of each study day and have been described previously (Nicholl et al., 2014).

### 2.4 Bioelectrical impedance analysis

BIA was measured using the RJL–BIA Sciences Quantum II system Cyprus 2.6 to assess body fat and fluid composition. Briefly, the RJL‐BIA passes an undetectable current of 9 V through the body (upper and lower) while participants recline supine on a table with their heads elevated at 45° for at least 10 min and their arms and legs comfortably abducted. Electrodes are placed in the standard tetrapolar lead distribution with two electrodes positioned on the dorsal side of the right hand and two on the dorsal side of the right foot with test completion within 5 min. RJL Systems' BIA devices use a 50 kHz signal to measure resistance (R, Ω) and reactance (Xc, Ω). Total body impedance (Z, Ω) and phase angle (°) are then calculated from the measured resistance and reactance. Resistance (R, Ω) is the opposition to the flow of an injected alternating current, at any current frequency, through intra‐ and extracellular ionic solutions (the components of TBW). In the human body, high resistance is associated with smaller amounts of FFM, whereas lower resistance is associated large amounts of FFM (Kyle et al., 2004a). Reactance (Xc, Ω) is the capacitive component of body cell mass (intracellular mass including cell membranes, organelles, and tissue interfaces) (Kyle et al., 2004a). In humans, high reactance is associated with large amounts of body cell mass, whereas low reactance is associated with smaller amounts of body cell mass. Resistance and reactance are both vector components of total body impedance (Z, Ω) with changes in impedance measurements reflecting overall changes in hydration and cell mass (Kyle et al., 2004a). A lower total body impedance is therefore considered to reflect an overall healthier state. Phase angle (°), reported in degrees, is the ratio between reactance and resistance (proportional to the ratio of body cell mass to FFM) and is a measure of how much the signal is delayed by the reactance (Kyle et al., 2004a). A higher phase angle is consistent with large quantities of intact cell membranes and body cell mass, whereas a lower phase angle is an indication of breakdown in the selective permeability of cellular membranes and consistent with an inability of cells to store energy. Thus, lower phase angles represent higher degrees of fluid overload. These bioimpedance measurements are then input into the BIA software along with the participants' age, sex, height, and weight to obtain (FFM, kg, and %BM), TBW (L and %FFM), TBW index (TBWI, L/m^2^), TBW fraction (L/kg), ICW (L and %TBW), and ECW (L and %TBW). The equations used by the RJL–BIA Sciences Quantum II software are based on the third National Health and Nutrition Examination Survey (NHANES‐III) data set (Chumlea et al., 2002; Sergi et al., 1994; Sun et al., 2003).

### 2.5 Anthropometric measurements

Weight (kg) was measured via digital scale in street clothes without outerwear. Standing height (cm) without shoes was measured with a tape measure. Body mass index (BMI) was calculated as weight/height^2^ (kg/m^2^). Standing abdominal circumference (cm) was measured between the last rib and the iliac crest. Hip circumference was measured at the maximum buttock circumference. Neck circumference (cm) was measured at the point just above the cricoid cartilage. FFM and fat mass (FM) were evaluated by BIA as described above. FFM was calculated by weight–FM. FM and FFM were adjusted for height ([FM or FFM]/height^2^) yielding FM Index (FMI) or FFM Index (FFMI), respectively.

### 2.6 CPAP therapy

After completing the first study day, participants were treated with CPAP as per published guidelines (Epstein et al., 2009). All participants underwent an auto‐CPAP trial to determine individual CPAP requirement. Initial auto‐CPAP settings were 16–6 cm H_2_O and were automatically titrated according to the CPAP unit titration algorithm to optimize therapy. If airflow limitation or NH were not fully corrected, participants were switched to fixed CPAP, which was estimated from the CPAP level at the 95th percentile. Adherence to CPAP therapy was monitored by electronic download from the unit each month. Once satisfactory CPAP adherence was achieved (defined as CPAP use for >4 h/night on >70% nights for 4 weeks; Epstein et al., 2009) and correction of OSA and NH was confirmed by a repeat HSAT while using CPAP, participants underwent reassessment of bioelectrical impedance parameters during a second identical study day. Our protocol was designed to ensure that once acclimatization to CPAP had been established, all subjects received a similar duration of effective CPAP therapy prior to the reassessment. Study participants wore their CPAP at home on the night prior to BIA testing. Participants then commuted to study lab and underwent the study measurements first thing in the morning.

### 2.7 Analyses

Data are reported as mean ± standard error or number (percentage), where appropriate. Sex‐stratified analyses were performed in keeping with Sex and Gender Equity in Research guidelines (Heidari et al., 2016). Our primary outcomes were sex differences in body fluid composition parameters as measured by bioimpedance in OSA participants before and after CPAP therapy. Comparisons between women and men were conducted using the Mann–Whitney test (non‐parametric testing). Pre‐ and post‐CPAP comparisons for women and men were made using the Wilcoxon signed‐rank test (non‐parametric testing). Sensitivity analyses were conducted excluding participants with controlled hypertension. All statistical analyses were performed with statistical software package SPSS V.26.0 (IBM). All analyses were two‐tailed with a significance level of 0.05.

## 3 RESULTS

### 3.1 Study enrollment

Forty‐six participants completed the first study day (15 women, 31 men). Twenty‐nine participants (10 women, 19 men) completed both study days and were included in the final analyses. One male participant ingested a single dose of candesartan (AngII‐receptor blocker) the morning of study Day 1; this participant was studied in an identical fashion post‐CPAP, including candesartan ingestion, to allow for comparison of pre‐post‐CPAP results. This participant was included in the final analysis as the outcomes of interest were sex differences in body fluid composition parameters as measured by bioimpedance in OSA participants before and after CPAP therapy.

### 3.2 Pre‐CPAP baseline characteristics

Participant baseline characteristics stratified by sex are presented in Table 1. Twenty‐nine (10 women [5 pre‐menopausal, 5 post‐menopausal], 19 men) newly diagnosed participants with OSA and NH were recruited. All participants were non‐diabetic, sodium‐replete, with normal kidney function, and BP <140/90, and without diurnal hypoxemia.

**TABLE 1:** Baseline characteristics by sex pre‐ and post‐CPAP therapy.

	Pre‐CPAP			Post‐CPAP		
	Women	Men	*p*‐value^b^	Women	Men	*p*‐value^b^
*N*	10	19	—	—	—	—
Age, years	50 ± 4	48 ± 2	0.5	—	—	—
Menopause, *N* (% women)	5 (50)	—	—	—	—	—
Race, *N* (% White)^c^	10 (100)	14 (74)	0.13	—	—	—
Hypertension, *N* (%)^f^	3 (30)	6 (32)	1.0	—	—	—
Daytime SaO_2_, %	94.4 ± 0.9	94.6 ± 0.4	1.0	—	—	—
Height, cm	164 ± 3	177 ± 1	0.001	164 ± 3	177 ± 1	0.001
Body mass, kg	104.0 ± 10.5	108.6 ± 4.1	0.2	104.3 ± 10.5	108.3 ± 3.7	0.16
BMI, kg/m^2^	38.2 ± 2.8	34.7 ± 1.3	0.4	38.6 ± 2.8	34.6 ± 1.1	0.2
Neck circumference, cm	38.9 ± 0.9	44.6 ± 0.7	<0.001	—	—	—
Neck: height	0.237 ± 0.005	0.252 ± 0.004	0.028	—	—	—
Abdominal circumference, cm	111.3 ± 5.3	114.4 ± 2.9	0.2	—	—	—
Abdominal: height	0.68 ± 0.03	0.65 ± 0.02	0.6	—	—	—
Hip circumference, cm	122.6 ± 4.2	114.4 ± 2.0	0.15	—	—	—
Hip: height	0.75 ± 0.02	0.65 ± 0.02	0.002	—	—	—
SBP, mm Hg	125 ± 4	127 ± 2	0.8	119 ± 4	123 ± 2	0.5
DBP, mm Hg	74 ± 3	80 ± 2	0.11	68 ± 2	76 ± 2^a^	0.017
MAP, mm Hg	91 ± 3	96 ± 2	0.4	85 ± 3	92 ± 2^a^	0.031
Heart rate, bpm	67 ± 4	68 ± 2	1.0	66 ± 3	64 ± 2	0.5
Serum creatinine, μmol/L	59 ± 3	82 ± 3	<0.001	61 ± 2	85 ± 3^a^	<0.001
eGFR, mL/min/1.73 m^2^	101 ± 3	96 ± 3	0.6	98 ± 2	93 ± 3^a^	0.4
Fasting glucose, mmol/L	4.7 ± 0.2	4.9 ± 0.1	0.7	5.0 ± 0.2	4.8 ± 0.1	0.5
Hemoglobin A1c, %	6.0 ± 0.1	5.9 ± 0.1	0.7	5.5 ± 0.4	5.8 ± 0.1	0.8
17β‐Estradiol, pmol/L	130 ± 44	109 ± 7	0.4	233 ± 69	100 ± 8	0.8
Pre‐menopausal	211 ± 73	—	0.064	332 ± 94	—	0.039
Post‐menopausal	49 ± 8^d^	—	0.005	96 ± 56^d^	—	0.095
Urine sodium, mmol/day	398 ± 21^e^	342 ± 29	0.073	380 ± 43^e^	335 ± 31	0.16

Abbreviations: BMI, body mass index; CPAP, continuous positive airway pressure; DBP, diastolic blood pressure; eGFR, estimated glomerular filtration rate, MAP, mean arterial pressure; SBP, systolic blood pressure.

^a^ *p* < 0.05 versus pre‐CPAP.

^b^ *p*‐value women versus men.

^c^ East Asian (*N* = 3), Mixed Origin (*N* = 1), South Asian (*N* = 1), White (*N* = 24).

^d^ *p* < 0.05 versus pre‐menopausal women.

^e^ *N* = 9.

^f^ Nine participants (three women, six men) were on antihypertensive medications. For women, antihypertensives included the following: angiotensin receptor blocker (*N* = 3) and thiazide‐like diuretic (*N* = 2). Two women were on dual therapy (angiotensin receptor blocker + thiazide‐like diuretic). For men, antihypertensives included the following: angiotensin‐converting enzyme inhibitor (*N* = 2), angiotensin receptor blocker (*N* = 2), beta‐blocker (*N* = 2), calcium channel blocker (*N* = 1), and thiazide‐like diuretic (*N* = 2). One man was on dual therapy (angiotensin receptor blocker + thiazide‐like diuretic) and one man was on quadruple therapy (angiotensin‐converting enzyme inhibitor, beta‐blocker, calcium channel blocker, and thiazide‐like diuretic).

Women and men had similar severity of OSA and nocturnal hypoxemia. As anticipated, men were taller than women, though there were no sex differences in body mass or BMI. Neck circumference and neck: height ratio were smaller in women than men. There were no differences in abdominal circumference, abdomen: height ratio, or hip circumference, and as expected hip: height ratio was greater in women than men. As expected, serum creatinine was lower in women than men, although there was no difference in eGFR. There were no sex differences in BP or glycemic parameters.

The baseline characteristics of those participants who completed the study compared to those who withdrew from the study are reported in Table 2. Withdrawn participants did not differ from participants who completed the study apart from having a higher SBP. Withdrawn participants numerically had more severe OSA, higher glycosylated hemoglobin, and greater BMI, though these differences were not statistically significant.

**TABLE 2:** Baseline characteristics of completed versus withdrawn participants.

	Completed	Withdrawn	*p*‐value^a^
*N*	29	17	—
Age, years	53 ± 2	49 ± 2	0.2
Sex, *N* (% women)	10 (34)	5 (29)	1.0
Menopause, *N* (% of women)	5 (50)	4 (80)	0.6
Race, *N* (% White)	24 (83)^b^	11 (65)^c^	0.3
Hypertension, *N* (%)	9 (31)	5 (29)	1.0
Height, cm	173 ± 2	171 ± 2	0.6
Body mass, kg	104.9 ± 4.9	113.9 ± 7.0	0.4
BMI, kg/m^2^	35.1 ± 1.4	38.4 ± 1.8	0.2
Neck circumference, cm	42.6 ± 0.7	44.0 ± 1.0	0.3
Neck: height	0.247 ± 0.005	0.257 ± 0.005	0.17
Abdominal circumference, cm	113.3 ± 4.1	118.5 ± 2.6	0.4
Abdominal: height	0.66 ± 0.01	0.70 ± 0.02	0.3
Hip circumference, cm	117.9 ± 2.0	122.7 ± 3.7	0.3
Hip: height	0.69 ± 0.01	0.72 ± 0.02	0.2
ODI, per hour	47.4 ± 4.4	59.9 ± 5.6	0.09
SaO_2_ <90, % monitoring time	48.3 ± 5.5	58.3 ± 6.3	0.3
Mean SaO_2_, %	88.3 ± 0.8	86.8 ± 0.9	0.06
Minimum nocturnal SaO_2_, %	68.8 ± 1.9	65.8 ± 1.9	0.12
Oximetry monitoring time, h	7.4 ± 0.3	7.2 ± 0.4	0.5
SBP, mm Hg	127 ± 2	138 ± 5	0.018
DBP, mm Hg	78 ± 2	79 ± 2	0.8
MAP, mm Hg	94 ± 2	99 ± 3	0.2
Heart rate, bpm	68 ± 2	66 ± 3	0.6
Serum creatinine, umol/L	59 ± 3	82 ± 3	0.5
eGFR, mL/min/1.73 m^2^	97 ± 2	91 ± 4	0.4
Fasting glucose, mmol/L	4.8 ± 0.1	5.0 ± 0.1	0.3
Hemoglobin A1c, %	5.9 ± 0.1	6.1 ± 0.1	0.07
Urine sodium, mmol/day	361 ± 21^d^	361 ± 34	1.0

Abbreviations: BMI, body mass index; CPAP, continuous positive airway pressure; DBP, diastolic blood pressure; eGFR, estimated glomerular filtration rate, MAP, mean arterial pressure; ODI, oxygen desaturation index; SaO_2_, oxyhemoglobin saturation; SBP, systolic blood pressure.

^a^ *p*‐value completed versus withdrawn.

^b^ East Asian (*N* = 3), Mixed Origin (*N* = 1), South Asian (*N* = 1), White (*N* = 24).

^c^ East Asian (*N* = 2), South Asian (*N* = 1), Southeast Asian (*N* = 1), Indigenous (*N* = 1), Latin American (*N* = 1), White (*N* = 11).

^d^ *N* = 28.

### 3.3 CPAP therapy

CPAP adherence parameters stratified by sex are reported in Table 3. All participants were adherent with CPAP, which corrected OSA and NH. CPAP was used 93% ± 2% of nights (84% ± 3% >4 h/night), with an average nightly usage of 6.4 ± 0.2 h, indicating excellent adherence, and a normal apnea‐hypopnea index (AHI), indicating adequate treatment. All participants met acceptable HSAT criteria for correction of OSA (ODI <10/h; Table 3) and all but five participants (three women, two men) met criteria for correction of NH (SaO_2_ ≥90% for <12% monitoring time); in four of five participants the post‐CPAP mean SaO_2_ was ≥90%. One participant had a mean SaO_2_ <90% (89% which improved from 85% pre‐CPAP). Two participants (one woman, one man) were unable to complete HSAT on therapy prior to reassessment. However, their CPAP downloads indicated an AHI 0.3 and 0.8/h with CPAP use >4 h/night 96% and 100%. Adequate therapy was inferred based on these data.

**TABLE 3:** Sleep characteristics pre‐ and post‐CPAP therapy and CPAP adherence parameters by sex.

	Pre‐CPAP			Post‐CPAP		
	Women	Men	*p*‐value^b^	Women	Men	*p*‐value^b^
*N*	10	19	—	—	—	—
ODI, per hour	39.9 ± 9.4	51.4 ± 4.4	0.085	2.9 ± 0.7^a^	3.4 ± 0.6^a^	0.7
SaO_2_ <90, % monitoring time	55.9 ± 12.3	44.3 ± 5.4	0.5	14.0 ± 7.2^a^ ^,^ ^c^	5.5 ± 1.7^a^ ^,^ ^d^	0.9
Mean SaO_2_, %	86.2 ± 2.0	89.3 ± 0.5	0.6	92.8 ± 0.8^a^ ^,^ ^c^	92.2 ± 0.3^a^ ^,^ ^d^	0.4
Minimum nocturnal SaO_2_, %	64.2 ± 4.5	71.2 ± 1.6	0.3	82.4 ± 2.6^a^ ^,^ ^c^	85.7 ± 0.8^a^ ^,^ ^d^	0.4
Oximetry monitoring time, h	7.5 ± 0.4	7.4 ± 0.4	0.8	7.4 ± 0.5^c^	6.9 ± 0.3^d^	0.4
Total days used, days	—	—	—	144 ± 20	145 ± 16	0.8
Average use/night, h	—	—	—	6.3 ± 0.4	6.4 ± 0.3	0.7
Use in 4‐week period prior to reassessment, % download time	—	—	—	93 ± 3	93 ± 2	0.7
Use >4 h/night in 4‐week period prior to reassessment, % download time	—	—	—	84 ± 5	83 ± 4	0.9
Apnea–hypopnea index, per hour	—	—	—	3.0 ± 1.5	2.7 ± 0.7	0.6

Abbreviations: CPAP, continuous positive airway pressure; ODI, oxygen desaturation index; SaO_2_, oxyhemoglobin saturation; SBP, systolic blood pressure.

^a^ *p* < 0.05 versus pre‐CPAP.

^b^ *p*‐value women versus men.

^c^ *N* = 9.

^d^ *N* = 18.

### 3.4 Body fluid and fat composition

Body fluid and fat composition parameters as measured by BIA pre‐ and post‐CPAP therapy are presented in Table 4. Pre‐CPAP, women had increased resistance compared to men though reactance was similar. Impedance was increased in women compared to men whereas phase angle was increased in men compared to women. Men had greater FFM parameters. TBW (%FFM) was similar between sexes, though absolute TBW (L), TBWI (L/m^2^), TBW fraction (L/kg) were increased in men. Women had greater ECW (%TBW) and reduced ICW (%TBW) compared to men. Consequently, the ECW: ICW ratio in women was greater than in men. The observed baseline sex differences in body fluid and fat composition parameters remained unchanged after CPAP therapy. Changes in body fluid and fat composition parameters in response to CPAP therapy did not differ by sex (Table 5).

**TABLE 4:** Body fluid and fat composition pre‐ and post‐CPAP therapy by sex.

	Pre‐CPAP			Post‐CPAP		
	Women	Men	*p*‐value^a^	Women	Men	*p*‐value^a^
*N*	10	19	—	—	—	—
Height, cm	164 ± 3	177 ± 1	0.001	164 ± 3	177 ± 1	0.001
Body mass, kg	104.0 ± 10.5	108.6 ± 4.1	0.2	104.3 ± 10.5	108.3 ± 3.7	0.16
BMI, kg/m^2^	38.2 ± 2.8	34.7 ± 1.3	0.4	38.6 ± 2.8	34.6 ± 1.1	0.2
Resistance (R), Ω	457.4 ± 25.4	388.5 ± 12.1	0.017	458.7 ± 23.1	385.0 ± 11.4	0.012
Reactance (Xc), Ω	53.2 ± 3.1	55.0 ± 3.1	1.0	55.1 ± 4.2	54.6 ± 3.2	0.9
Impedance (Z), Ω	460.5 ± 25.6	403.2 ± 15.4	0.048	462.0 ± 23.4	389.0 ± 11.6	0.015
Phase angle, °	6.7 ± 0.3	8.0 ± 0.3	0.005	6.8 ± 0.3	8.0 ± 0.3	0.011
Fat‐free mass, %BM	56.3 ± 1.7	71.7 ± 1.5	<0.001	56.8 ± 1.6	71.0 ± 1.6	<0.001
Fat‐free mass, kg	57.4 ± 4.2	77.0 ± 2.2	<0.001	58.4 ± 4.7	76.1 ± 1.9	0.002
FFMI, kg/m^2^	21.2 ± 1.0	24.6 ± 0.8	0.012	21.4 ± 0.9	22.8 ± 0.7	0.4
Fat mass, %BM	43.7 ± 1.7	28.3 ± 1.5	<0.001	43.2 ± 1.6	29.0 ± 1.6	<0.001
Fat mass, kg	46.6 ± 6.5	31.5 ± 2.5	0.017	46.0 ± 6.2	32.1 ± 2.6	0.031
FMI, kg/m^2^	17.1 ± 1.9	10.1 ± 0.8	0.001	16.9 ± 1.7	10.2 ± 0.8	0.002
TBW, %FFM	74.6 ± 0.4	74.3 ± 0.2	0.14	73.7 ± 0.9	74.9 ± 0.7	0.7
TBW, L	42.7 ± 3.0	57.2 ± 1.6	<0.001	42.7 ± 3.0	57.0 ± 1.4	0.001
TBWI, L/m^2^	15.8 ± 0.8	18.3 ± 0.6	0.013	15.9 ± 0.8	18.3 ± 0.5	0.017
TBW fraction, L/kg	0.42 ± 0.01	0.53 ± 0.01	<0.001	0.42 ± 0.01	0.53 ± 0.01	<0.001
ECW, %TBW	49.7 ± 0.7	44.0 ± 0.9	<0.001	49.6 ± 0.7	43.8 ± 0.5	<0.001
ECW, L	21.3 ± 1.7	25.2 ± 0.8	0.006	21.3 ± 1.8	25.0 ± 0.7	0.008
ICW, %TBW	49.7 ± 0.5	55.8 ± 0.9	<0.001	50.4 ± 0.7	56.3 ± 0.5	<0.001
ICW, L	21.1 ± 1.2	31.9 ± 1.0	<0.001	21.4 ± 1.2	32.1 ± 0.8	<0.001
ECW: ICW	1.00 ± 0.02	0.80 ± 0.04	<0.001	0.99 ± 0.03	0.78 ± 0.02	<0.001

Abbreviations: BM, body mass; BMI, body mass index; CPAP, continuous positive airway pressure, ECW, extracellular water; FFMI, fat‐free mass index; FMI, fat mass index; ICW, intracellular water; TBW, total body water.

^a^ *p*‐value women versus men.

**TABLE 5:** Changes in body fluid and fat composition in response to CPAP therapy by sex.

	Women	Men	*p*‐value^a^
*N*	10	19	—
**∆**Body mass, kg	0.3 ± 1.4	−0.3 ± 2.7	0.8
**∆**BMI, kg/m^2^	0.3 ± 0.6	−0.1 ± 0.9	0.4
**∆**Resistance (R), Ω	1.3 ± 11.4	−3.5 ± 5.4	0.6
**∆**Reactance (Xc), Ω	1.9 ± 2.5	−0.5 ± 1.0	0.6
**∆**Impedance (Z), Ω	1.5 ± 11.3	−14.3 ± 12.5	0.5
**∆**Phase angle, °	0.2 ± 0.3	0.0 ± 0.1	0.7
**∆**Fat‐free mass, %BM	0.5 ± 1.1	−0.6 ± 1.0	0.8
**∆**Fat‐free mass, kg	1.0 ± 1.4	−0.9 ± 1.7	0.9
**∆**FFMI, kg/m^2^	0.2 ± 1.7	−1.8 ± 1.0	0.13
**∆**Fat mass, %BM	−0.5 ± 1.1	0.6 ± 1.1	0.8
**∆**Fat mass, kg	−0.7 ± 1.2	0.6 ± 1.6	0.6
**∆**FMI, kg/m^2^	−0.2 ± 0.5	0.2 ± 0.5	0.7
**∆**TBW, %FFM	−1.0 ± 0.8	0.7 ± 0.7	0.14
**∆**TBW, L	0.0 ± 0.8	−0.2 ± 0.9	0.6
**∆**TBWI, L/m^2^	0.1 ± 0.3	0.0 ± 0.3	0.7
**∆**TBW fraction, L/kg	0.00 ± 0.01	0.00 ± 0.01	0.8
**∆**ECW, %TBW	−0.1 ± 0.8	−0.3 ± 1.0	0.3
**∆**ECW, L	0.0 ± 0.3	−0.2 ± 0.6	0.4
**∆**ICW, %TBW	0.7 ± 0.4	0.5 ± 1.0	0.2
**∆**ICW, L	0.3 ± 0.4	0.1 ± 0.7	0.7
**∆**ECW: ICW	−0.01 ± 0.02	−0.02 ± 0.04	0.4

Abbreviations: BM, body mass; BMI, body mass index; CPAP, continuous positive airway pressure, ECW, extracellular water; FFMI, fat‐free mass index; FMI, fat mass index; ICW, intracellular water; TBW, total body water.

^a^ *p*‐value women versus men.

## 4 DISCUSSION

We utilized BIA technology to evaluate sex differences in body fluid composition in women and men with symptomatic OSA before and after initiation of CPAP therapy. To our knowledge this is first study to utilize BIA to examine sex differences in body fluid composition in participants with OSA and normal kidney function before and after treatment with CPAP. Our primary findings were: (1) pre‐CPAP, despite TBW (%FFM) being similar between sexes, women had increased ECW (%TBW) and reduced ICW (%TBW) compared to men; (2) women also had increased resistance, increased impedance, and reduced phase angle compared to men; and (3) the response of body composition parameters to 1 month of CPAP therapy did not differ by sex. Our findings support that there are underlying sex differences in body fluid composition in OSA, though contrary to our expectations, women with OSA in our study had body fluid and fat composition parameters which favored volume expansion (increased ECW, reduced phase angle) compared to men. Importantly, the impact of CPAP therapy on body fluid composition did not differ by sex. Our data does not prove that the persistent sex differences in body composition following treatment with CPAP are responsible for the pathogenesis of OSA in women as CPAP may correct OSA independently of changes in body fluid composition.

There is a growing body of research utilizing BIA to evaluate body fat and fluid composition in OSA (Kim et al., 2021; Kosacka et al., 2013; Oğretmenoğlu et al., 2005); however, few studies have reported sex‐stratified results. Kosacka et al. studied 127 patients with OSA (27% female) and found that patients with OSA had increased BMI, TBW, and %ECW, and decreased %ICW and phase angle compared to control participants (Kosacka et al., 2013). The authors reported positive correlations between AHI and BMI and with %ECW and inflammatory markers, while negative correlations were observed between AHI and %ICW (Kosacka et al., 2013). Other studies have demonstrated that OSA severity correlated with body composition parameters as measured by BIA more strongly than general and cervical obesity (Lovin et al., 2010; Oğretmenoğlu et al., 2005). Kim et al. utilized BIA in 2064 patients with OSA (22.9% female) from Korea to evaluate sex differences in body fat and muscle composition (Kim et al., 2021). The authors found that in male patients with OSA, both BIA fat and muscle composition were correlated with AHI, whereas in female patients with OSA fat indicators were correlated with AHI, while muscle indicators were not (Kim et al., 2021). This study revealed different patterns in body fat and muscle composition in OSA depending on sex (Kim et al., 2021), which may account for the established sex differences in symptomology, pathogenesis, and severity in OSA (O'Connor et al., 2000). Our study extends on the work of previous studies by demonstrating that women with severe OSA have increased ECW and reduced ICW despite similar TBW (%FFM), as well as reduced phase angle, compared to men.

Fluid retention contributes to the pathogenesis of OSA through nocturnal rostral fluid shift from the lower extremities to the neck overnight resulting in upper pharyngeal narrowing and worsening OSA severity in otherwise healthy adults and populations prone to aberrant volume redistribution, such as CHF and end‐stage kidney disease (ESKD) (Bucca et al., 2007; Ding et al., 2014; Elias et al., 2012; Kasai et al., 2012; Redolfi et al., 2009; Silva et al., 2017), although it was not reported if the results differed by sex. In patients with CHF and severe OSA diuretic therapy improved OSA severity and reduced pharyngeal caliber (Bucca et al., 2007), while patients with ESKD on peritoneal dialysis, pharyngeal edema was associated with new onset severe OSA (Tang et al., 2009). In patients with ESKD maintained on hemodialysis, fluid overload influences overnight rostral fluid shift and OSA severity the night before and after hemodialysis (Ogna et al., 2015) while ultrafiltration removal of ~2 L of fluid reduced the AHI in patients with OSA by 36% (Lyons et al., 2017). Following kidney transplantation there is a significant reduction in TBW and fluid overload volume accompanied by a reduction in OSA severity compared to control participants who remained on dialysis (Forni Ogna et al., 2020). However, in one small study of predominantly men with advanced Stage IV CKD or ESKD maintained on hemodialysis, TBW as a proportion of body size as determined by BIA was not predictive of OSA severity (Huang et al., 2017). Finally, in non‐obese otherwise healthy men, larger baseline neck circumference and BIA phase angle of the neck, which has been associated with larger pharyngeal tissue content, was associated with increased susceptibility to worsening OSA in response to fluid overload (Gavrilovic et al., 2016).

Lower fluid accumulation at night in the necks of women may to contribute to reduced OSA severity compared to men (Elias et al., 2012; Kasai et al., 2012; Su et al., 2009). Rostral fluid displacement by lower‐body positive pressure increased upper airway collapsibility more in healthy, non‐obese men than women (Su et al., 2009). Similarly, in patients with CHF and OSA and patients with ESKD maintained on hemodialysis, studies of the nocturnal rostral fluid shift have demonstrated a significant relationship between OSA severity and the change in leg fluid volume to the neck in men but not in women (Elias et al., 2012; Kasai et al., 2012). In our study we found that women with OSA had increased ECW and reduced ICW compared to men despite similar TBW (%FFM) and severity of OSA and nocturnal hypoxemia. We speculate that despite the increased ECW observed in women with OSA, which may be thought to predispose to fluid overload, increased upper airway narrowing and increased OSA severity, that, similar to previous studies, women with OSA have reduced nocturnal rostral fluid shift and a volume redistribution which favors other parts of the body away from the neck, such as the torso (Silva et al., 2017).

OSA promotes metabolic syndrome by impacting parameters involved in energy balance regulation including abnormal dysregulation of hunger/satiety, increased food intake, increased energy metabolism, and reduced physical activity (Shechter, 2016). Weight loss is known to improve OSA, yet CPAP is often associated with weight gain (Drager et al., 2015). A meta‐analysis of randomized control trials has demonstrated that OSA treatment with CPAP therapy promotes an increase in both weight and BMI (Drager et al., 2015). Another study concluded that CPAP had no effect on visceral fat while others have reported CPAP therapy to increase lean body mass in both sexes (Münzer et al., 2010). CPAP appears to reduce abnormally high ghrelin and leptin levels, thereby reducing excess food intake, and alter energy metabolism by reducing resting and sleep metabolic rates, but does not appear to have a significant impact on physical activity (Shechter, 2016).

While previous studies have focused on the impact of CPAP on body fat/muscle composition, there is a paucity of research utilizing BIA to evaluate the effect of CPAP on body water composition in OSA. These studies have been small, not utilized sex‐stratified analyses or excluded women altogether, or have found conflicting results. Hence, whether there are sex differences in body fat and fluid composition as determined by BIA in the OSA population and particularly after CPAP therapy remains unknown. In a double blind, randomized control trial of 42 men with type‐2 diabetes and newly diagnosed OSA who underwent either therapeutic or placebo CPAP for 3 months there was no significant difference in BMI, bioimpedance, or anthropometric measures (West et al., 2007). In another small open label, non‐randomized trial of CPAP versus no treatment in 24 patients with OSA (42% female) which examined the effect of CPAP on total body composition as measured by air displacement plethysmography found that after 8 weeks of CPAP there was an increas in body weight and FFM, suggesting that the weight gain is driven by increases in lean body mass (Shechter et al., 2019), but whether this finding differed by sex was not reported. A previous meta‐analysis has demonstrated that OSA treatment with CPAP promotes an increase in both weight and BMI (Drager et al., 2015). Finally, Silva et al. compared the effects of compression stockings (CS) and CPAP on fluid dynamics in patients with OSA on hemodialysis (Silva et al., 2017). The authors found that CS reduced OSA severity by avoiding fluid retention in the legs, favoring accumulation in the intracellular component of the trunk, thus avoiding fluid shift to the neck, while CPAP improved OSA by exerting local pressure on the upper airway, with no impact on fluid redistribution (Silva et al., 2017). Overall CPAP performed better than CS for both reduction of AHI and overnight reduction of neck circumference (Silva et al., 2017).

Whole body bioimpedance parameters of fluid overload appear to be consistently and positively correlated with increased risk of death and adverse cardiovascular outcomes (Mayne et al., 2022). Liu et al. reported that every increment increase in ECW (%TBW) by 0.001 was associated with an increased risk of a heart failure‐related event (hospitalization or death) by 6% (HR 1.06, 95% CI 1.02–1.10) in patients with CKD (Liu et al., 2012). Ohashi et al. reported that per increment increase of ECW:ICW was associated with an increased risk for hospitalization, 50% decline in GFR or renal replacement therapy, and all‐cause mortality (Ohashi et al., 2015). There has also been growing attention to the significance of tissue impedance measures including resistance, reactance, and phase angle, each of which may vary based on tissue composition (de Luis et al., 2010; Kyle et al., 2004b). Decreased resistance and total body impedance and increased reactance and phase angle have been associated with increased tissue hydration, body cell mass, and FFM (Baumgartner et al., 1988; Kyle et al., 2004b). Lower phase angle has been associated with reduced muscle mass and increased mortality in several patient populations including HIV‐infected, ESKD on dialysis, CHF, and liver cirrhosis (Beberashvili et al., 2014; Kyle et al., 2004b), and has been proposed to be a novel cardiovascular risk factor (de Luis et al., 2010). Caravaca et al. reported that each degree reduction in phase angle was associated with increased mortality (HR 0.49, 95% CI 0.26–0.92) in patients with CKD (Caravaca et al., 2011). In another study, Bansal et al. reported a reduced phase angle <5.59 was associated with a double risk of all‐cause mortality compared to those with a phase angle ≥6.4 (Bansal et al., 2018). It should be noted that in our study while women had a reduced phase angle compared to men this was still greater than reduced phase angle reported in previous studies to be associated with increased mortality (Kyle et al., 2004b). Taken together, our findings support that women with OSA have expanded extracellular fluid, reduced tissue hydration, and reduced lean mass compared to men with OSA, which may contribute to sex differences in the pathogenesis of OSA through differences in body composition (Harik‐Khan et al., 2001). CPAP therapy for 1 month does not appear to impact body composition in either sex.

Our study has strengths and limitations. First, our study was limited to participants with OSA without co‐morbidities (other than controlled hypertension and obesity), limiting the generalizability of our results. Our sample size was relatively small and included fewer female participants, raising the possibility that our exploratory study was underpowered to detect true effects. Our study participants were also recruited from community patients referred for suspected OSA. Consequently, our study population represents a symptomatic OSA population, and may not be generalizable to an asymptomatic OSA population. There also remains the possibility that the study may have been prone to a healthy volunteer selection bias. However, by studying a healthier population, we were able to examine sex differences in body fluid composition and the response to CPAP in OSA without confounding factors and we were able to recruit a sufficient sample size of women and men to be able to conduct sex‐stratified analyses. Second, our study did not include control groups of participants without OSA, participants with OSA that were non‐adherent to CPAP, or participants with OSA who received either no CPAP or sham‐CPAP therapy. We attempted to minimize the effect of sample size and intra‐individual variability by utilizing a homogenous study group with careful pre‐study design. While no control group was included, conditions during the assessment were standardized to minimize the impact of potential confounders. Given the absence of healthy control group, we are unable to comment on whether the difference in distribution of body water between women and men observed in our study is specific to OSA or whether it is also present in healthy populations. Third, as we conducted total body BIA, specific body compartments such as pharyngeal and leg fluid composition was not directly assessed and hence the mechanistic relationship cannot be confirmed. It remains possible that CPAP may have a localized effect on body water distribution in the cervical region. Fourth, while BIA is a reliable method to assess body composition there are some limitations in the morbidly obese (BMI >34) (Coppini et al., 2005). Obese individuals have a relatively increased amount of TBW and ECW which may overestimate FFM and underestimate FM (Coppini et al., 2005). While this limitation may impact our study results, given we evaluated the same participants serially using the same technique, any differences in the estimation of effect size due to obesity should have been similar on both study days. Fifth, the duration that patients used CPAP prior to reassessment of body composition parameters varied due to differences in the ability of individual patients to acclimatize to CPAP, which is well‐recognized in patients with OSA (Hanly & Pierratos, 2001; Wadhwa & Mendelson, 1992). Further, the treatment duration of 4 weeks of effective CPAP therapy prior to reassessment may have been an insufficient duration to observe differences in body composition parameters. However, it was important to standardize the duration of efficacious intervention to determine the effect of CPAP on bioimpedance parameters and the treatment period we chose has been shown to improve cardiovascular measures in previous studies (Faccenda et al., 2001). We are thus unable to comment on the potential effects of longer exposure to CPAP therapy on body composition and it is entirely possible that outcomes may vary with a longer or less variable duration of therapy. Sixth, participants were studied the morning after CPAP use and not while wearing CPAP, which may also account for the lack of observed changes with CPAP therapy. Seventh, we used portable monitoring instead of polysomnography both to diagnose OSA and evaluate patients' responses to CPAP. This was appropriate for our study population according to guidelines (Epstein et al., 2009) and the findings were objective and unequivocal. Finally, gender identity was self‐reported by study participants, and we did not determine sex by karyotype. However, we measured sex hormone levels in all participants and given that no participant was taking exogenous sex hormones, we are confident that all participants were cis‐gender and that our results represent true sex differences in body composition.

In this community‐based population of women and men who were referred with symptomatic OSA and normal kidney function, we found sex differences in body fluid composition as determined by BIA. Women had expanded ECW, reduced ICW, and reduced phase angle compared to men. Importantly, the impact of CPAP therapy for 1 month on body composition did not differ by sex. While our study was small and exploratory in nature, it remains possible that sex differences in body fluid composition observed may still contribute to the pathogenesis of OSA, though CPAP therapy appears to correct OSA independently of changes in body composition. Larger prospective studies with greater numbers of pre‐ and post‐menopausal women and appropriate control groups are needed to confirm our findings.

## AUTHOR CONTRIBUTIONS

*Study design*: David D. M. Nicholl, Patrick J. Hanly, Sofia B. Ahmed. *Acquisition, analysis, data interpretation, and review of the manuscript for important intellectual content*: David D. M. Nicholl, Patrick J. Hanly, Jennifer M. MacRae, Ann A. Zalucky, George B. Handley, Darlene Y. Sola, Sofia B. Ahmed. *Drafting of the manuscript*: David D. M. Nicholl, Patrick J. Hanly, Sofia B. Ahmed. All authors have seen and approved the manuscript.

## FUNDING INFORMATION

This study was funded by an Establishment Grant from Alberta Innovates–Health Solutions. Sofia B. Ahmed is supported by Alberta Innovates–Health Solutions and the Canadian Institute of Health Research. Funding sources had role in study design, reporting, or reporting.

## ETHICS STATEMENT

The study protocol was approved by the Conjoint Health Research Ethics Board at the University of Calgary. Written informed consent was obtained from all study participants in accordance with the Declaration of Helsinki.

## CONFLICT OF INTEREST STATEMENT

The authors have no conflict of interest to disclose.
